# Rhein Suppresses Lung Inflammatory Injury Induced by Human Respiratory Syncytial Virus Through Inhibiting NLRP3 Inflammasome Activation *via* NF-κB Pathway in Mice

**DOI:** 10.3389/fphar.2019.01600

**Published:** 2020-01-28

**Authors:** Cunsi Shen, Zhengguang Zhang, Tong Xie, Jianjian Ji, Jianya Xu, Lili Lin, Jing Yan, An Kang, Qigang Dai, Yingmei Dong, Jinjun Shan, Shouchuan Wang, Xia Zhao

**Affiliations:** ^1^Jiangsu Key Laboratory of Pediatric Respiratory Disease, Institute of Pediatrics, Affiliated Hospital of Nanjing University of Chinese Medicine, Nanjing, China; ^2^School of Medicine & Holistic Integrative Medicine, Nanjing University of Chinese Medicine, Nanjing, China; ^3^Jiangsu Engineering Research Center for Efficient Delivery System of TCM, College of Pharmacy, Nanjing University of Chinese Medicine, Nanjing, China

**Keywords:** rhein, respiratory syncytial virus infection, lung inflammation, pro-inflammatory cytokine, tissue damage

## Abstract

Rhein is one of active anthraquinone components in traditional Chinese herbal medicine *Rheum palmatum L.*, possessing anti-inflammatory, antioxidant, antitumor, antiviral, and hepatoprotective activities. Human respiratory syncytial virus (RSV), a common virus, is able to result in pneumonia and bronchitis, which usually can be seen in infants. However, so far the effects of Rhein on RSV-induced pneumonia are still unknown. As the NLRP3 inflammasome is activated excessively, it is able to lead to inflammatory response and tissue injury in most viral infection process (including RSV infection) of respiratory tract. Therefore, we designed experiments to reveal whether Rhein can treat RSV-induced pneumonia by inhibiting NLRP3 inflammasome activation. In present research, we established the pneumonia model of BALB/C mice caused by RSV. First of all, the pathology of lung tissue and the weight of mice were evaluated, and the corresponding lung index was calculated. Additionally, the expression of pro-inflammatory mediators in serum and lung tissues, and related proteins (NLRP3, ASC and Caspase-1) of NLRP3 inflammasome and NF-κB pathway were detected by Enzyme-linked immunosorbent assay (ELISA), Real-time PCR (RT-PCR), Immunohistochemistry (IHC), and Western blot (WB), respectively. The determination of lung index and lung tissue pathological evaluation revealed that Rhein was able to alleviate lung infection and injury caused by RSV. The results of ELISA showed that Rhein was able to reduce the release of pro-inflammatory cytokines in the serum and lung tissues of RSV-induced BALB/c mice, including IL-1β, IL-6, TNF-α, IL-18, and IL-33. Additionally, it was revealed that Rhein inhibited the immune inflammatory response of RSV-infected mice, which was likely to be associated with the inhibition the NLRP3 inflammasome activation *via* NF-κB pathway. To sum up, our results indicated that Rhein may inhibit RSV-induced pulmonary inflammatory response effectively; meanwhile, it is emphasized that Rhein therapy is likely to be a promising treatment on the RSV-infected lung inflammation and avoidance of lung tissue damage.

## Introduction

As a major reason of respiratory infection of infants and children, human respiratory syncytial virus (RSV) infection is also one of the main clinical symptoms of pneumonia and bronchiolitis (Aranda and Polack, 2019). In prophylactic treatment of RSV infection, palivizumab was commonly used, and it is a neutralized monoclonal antibody, which is generally used in RSV infection of high-risk infants with RSV (Rezaee et al., 2017; Acero-Bedoya et al., 2019). Nevertheless, lung histopathology and inflammation are not able to be improved by the antibody (Olszewska et al., 2011). Ribavirin is given the permission to treat virus infection extensively, including all infants and immunocompromised patients with serious infection with RSV. However, the effectiveness of ribavirin is extremely limited, and it has more additional side effects. For example, it may cause hemolytic anemia (Krilov, 2011; Mooney et al., 2014). Therefore, it is important to identify good therapeutic strategies to treat the inflammation and lung tissue damage caused by RSV.

It remains unknown of the precise pathogenetic mechanisms of RSV that causes disease. The main clinical expression of RSV infection is excessive inflammatory and tissue damage (Lee et al., 2017; Vázquez et al., 2019). The release of pro-inflammatory cytokines usually causes excessive inflammatory response, mainly including interleukin IL-1β, IL-6, tumor necrosis factor (TNF-α) (Sun et al., 2018), and other members of the IL-1 gene family, for example, IL-18, IL-33 (Han et al., 2017; Yaneisi et al., 2019), etc. The Pulmonary inflammation and injury maybe caused by inflammasome activation, which can be resulted from viral infection of RSV infection (Yoo et al., 2013; Triantafilou and Triantafilou, 2014; Rivera-Toledo et al., 2017; Segovia et al., 2017). Activation of the inflammasome has a strong impact on RSV infection and is able to initiate the innate immune defense function, activation of Caspase-1, as well as the maturation of related inflammatory factors, such as IL-1β, IL-18, and IL-33 while NLRP3 is closely related to the development of many inflammatory diseases and is one of the most important types of inflammasome. Previous studies have shown that NF-κB is an important factor in the activation of NLRP3 inflammasome during RSV infection and there is a positive correlation between them. Additionally, pro-inflammatory cytokines, such as IL-1β, etc, as target genes of NF-κB, can also be transcriptional activated by NF-κB (Segovia et al., 2017).

In the academic sector of anti-inflammatory and anti-viral pharmacology, the NLRP3 inflammasome and NF-κB signaling both play essential role in the excessive inflammatory response stimulated by various types of viruses, including RSV, and are closely related to the viral-induced lung injury (Xu et al., 2015; Tate et al., 2016; Chen et al., 2017; Coates et al., 2017; Pinar et al., 2017; Ren et al., 2017; Sarvestani and McAuley, 2017). As for the application of effective treatment strategies against inflammatory response caused by RSV invasion, there has been no research paradigm until now. On the basis of previous studies, we have designed some experiments to explore whether inhibiting NLRP3 inflammasome activation *via* NF-κB pathway can reduce viral infection and lung injury caused by RSV. In fighting virus infection to save patients, traditional Chinese medical science was in major position; in the meantime, phytotherapy has a long historical standing in China, Korea, Japan, and India. In the field of clinical research, it is generally known that Traditional Chinese Medicine (TCM) has abundant resources, and it has the advantages; for example, low toxicity, high efficiency (Wang and Liu, 2014), etc. Currently, traditional Chinese medicine therapy is indeed considered to be an effective method to combat inflammation caused by viral infection, and many Chinese herbs have been confirmed to have antiviral effect. Screening efficient antiviral compounds from Chinese herbal medicine is not only beneficial, but also an effective way to find new antiviral drugs (Huang et al., 2014). Additionally, there are also reports finding that the ethanolic extract of rhubarb is able to inhibit virus infection, actually (Lin et al., 2016). Anthraquinone, the major active constituent of the crude extract of rhubarb, covers several compounds such as Rhein, Emodin, Chrysophanol, Physcion, Aloe-emodin, etc. Furthermore, in some other TCMs like *Polygonum cuspidatum Sieb.et Zucc, Aloe barbadensis Miller, Cassia angustifolia Vahl*, and *Polygonum multiflorum Thunb*, Rhein also exists universally.

**Figure 1:**
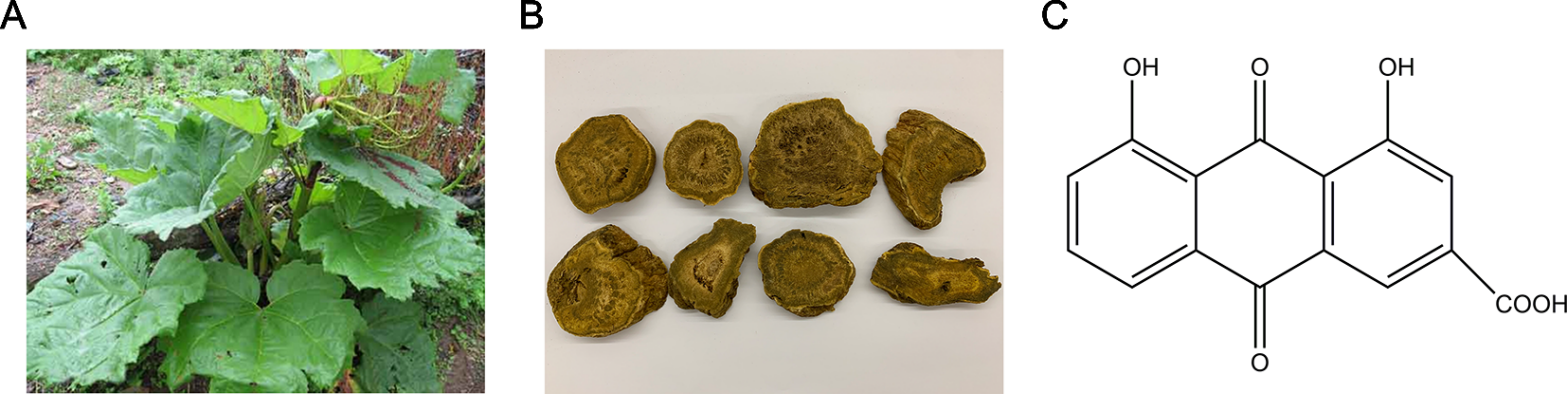
**(A)** The plant source (*Rheum palmatum L*.) of Rhein (C_15_H_8_O_6_). **(B)** The rhizome of *R. palmatum L*. containing Rhein. **(C)** The chemical structure of Rhein.

Rhein comes from the rhizome of *Rheum palmatum L*. and is deemed to be provided with high electrochemical oxidoreduction activity (**Figure 1**). It possesses two hydroxide residues, one carboxyl, and has very strong chemical polarity (Malterud et al., 1993). Although Rhein has antioxidant, antiviral, anti-inflammatory, antitumor, anti-fibrosis, hepatoprotective, and nephroprotective activities (Sun et al., 2016; Bi et al., 2018), the anti-RSV pneumonia of Rhein has not yet been clearly defined. Furthermore, previous literature studies found that Rhein had the anti-inflammatory effect through intervention NLRP3 signal in RAW264.7 macrophages, and zebrafish (Ge et al., 2017). On the basis of these literature review, We built a RSV-induced pneumonia model in BALB/c mice for this experiment, and in the course of this study, we not only observed the inhibitory effect of Rhein on pneumonia induced by RSV infection, but also thought whether it deserves to further study, and whether the NLRP3 inflammasome and NLRP3 inflammasome activation *via* NF-κB pathway were involved in the related molecular mechanisms.

## Materials and Methods

### Materials

Rhein (C_15_H_8_O_6_, purity > 98%, #110757) was obtained from National Institutes for Food and Drug Control (Beijing, China). Ribavirin was purchased from Sigma (USA). Fetal bovine serum and Dulbecco’s Modified Eagle’s medium (DMEM) were obtained from Gibco (USA). Human laryngeal epithelial carcinoma cell line (HEp-2) was obtained from Shanghai Institute of Biochemistry and Cell Biology (Shanghai, China). Human RSV strain was obtained from Institute of viruses, Wuhan University (Wuhan, China). Penicillin–streptomycin solution was obtained from Sigma (100U/mL) (USA).

### Protocol of Animal Experiments *In Vivo*

The 6-week-old female BALB/c mice of SPF grade were obtained from Comparative Medicine Centre of Yangzhou University (Yangzhou, China) [No. SCXK (Su) 2017–0007]. The animals were raised for 3 days for acclimation with 12-hour light-dark cycles; meanwhile, animals were also given plenty of clean pellet feed and water. In the course of the experiment, mice were assigned randomly to six groups (n = 10): the control group, the RSV-induced group, Rhein treatment groups at three different concentrations (30mg/kg/day, 60mg/kg/day, and 120mg/kg/day) and the positive control group (ribavirin) (46mg/kg/day). Mice were slightly anesthetized with ether nasal drops and given nasal drops with RSV virus solution [6.8×10^6^ plaque forming units (PFU)]. After RSV stimulation daily, mice in positive control group and Rhein treatment groups were given ribavirin and Rhein for 5 days consistently by using the method for intragastric administration. For the mice in RSV-induced group and control group, we gave them the same dose of saline in place of the drug. Additionally, we gave the mice in normal control group culture supernatant of Hep-2 cell lysate with negative RSV infection after centrifugation (the centrifugation condition was the same as virus suspension). We weighed the mice for 5 days and at the end of the experiment, we dissected each group of mice and removed the lung tissues. As it is shown in the following formula, we calculated the lung index: lung index = lung weight(g)/body weight (g)×100%. Based on the National Institutes of Health Guidelines for Laboratory Animals and approved by the Animal Ethics Committee of Nanjing University of Chinese Medicine, we performed all steps of laboratory.

### Enzyme-Linked Immunosorbent Assay (ELISA)

We evaluated the effect of Rhein on inflammatory cytokines released in mice infected with RSV by estimating the concentration of IL-1β, IL-6, TNF-α, IL-18, and IL-33 in the lung tissues and serum using commercially available ELISA kits (MULTI SCIENCES, China). We performed all steps of experiment according to the protocol provided of the manufacturer.

### Real-Time PCR

The lung tissues were homogenized and the total RNA was extracted using Trizol (Takara, China),then the RNA was reverse transcribed into cDNA using the Prime Script^®^ RT reagent kit (Takara, China). Real-time PCR assay on the samples was carried out using the SYBR^®^ Premix Ex Tap™ II (Takara, China) as follows: The conventional reaction conditions for Real-time PCR assay were as follows: one cycle of 95°C for 30s, followed by 40 cycles of 95°C for 5s and 60°C for 34s. All experiments were repeated for three times. The Real-time PCR data were analyzed by QuantStudio 7 Flex detection system (Applied Biosystems Co., USA). The 2^−ΔΔCt^ method was used to evaluate the mRNA levels of specified genes after normalization by GAPDH. The specific primer sequences were used in **Table 1**.

**Table 1:** The specific primers used in RT-PCR assay.

Gene name	Forward (5′-3′)	Reverse (5′-3′)
NLRP3	CATCAATGCTGCTTCGACAT	TCAGTCCCACACACAGCAAT
IL-1β	GCTGCTTCCAAACCTTTGAC	AGCTTCTCCACAGCCACAAT
ASC	AGACCACCAGCCAAGACAAG	CTCCAGGTCCATCACCAAGT
Caspase-1	CACAGCTCTGGAGATGGTGA	GGTCCCACATATTCCCTCCT
GAPDH	TGGCCTTCCGTGTTCCTAC	GAGTTGCTGTTGAAGTCGCA

### Histopathological and Immunohistochemical Detection

After the experiment, the lung tissues were harvested. Lobules of lung were randomly selected from the mice, and each sample was fixed into 4% paraformaldehyde, and then dehydrated. Further, the samples of each group were paraffin-embedded and cut into 3-μm thick sections. The sections were then stained with hematoxylin-eosin and examined with light microscopy. Two pathologists who were not aware of sample assignment to experimental groups scored the lung sections. The degree of pathological changes was represented as the mean of ten different fields in each section classified at the level of 0–3 (0: normal, 1: mild, 2: moderate, 3: severe). Then, some other sections of each group were used for immunohistochemical experiments, followed by evaluating the expression of NLRP3 or IL-1β (Abcam Ltd.). The IHC positive results were expressed as brown staining in cytoplasm and we used Image-Pro Plus 6.0 software to quantify as average optical density (AOD) [AOD  =  Integrated Optical Density (IOD) SUM/Area SUM] (Jin et al., 2017).

### Western Blot Analysis

By using RIPA lysate containing phosphatase inhibitor PhosStop and protease inhibitor PMSF, we extracted total protein of lung samples. In order to remove debris, we centrifuged the lysed samples for 30 min at 13,000g. The protein concentrations of each lung sample in different groups were measured by using a BCA protein concentration assay kit. The content of total protein was14μg mixed with 2X electrophoresis sample buffer (Bio-Rad, USA), denatured at 100°C for 5 min, and then they were stored at −80°C. Total proteins were separated by 8–12% SDS-PAGE. The separated proteins were transferred to a polyvinylidene fluoride (PVDF) membrane after electrophoresis, (0.22 or 0.45μm, Millipore, USA), blocked with 5% BSA in TBST for 2h, then incubated with designated first antibodies, including NF-κB (P65), p-NF-κB (P65), IκBα, p-IκBα, NLRP3, ASC, Caspase-1 (P20), IL-1β, β-actin, and Histone H3 (Abcam Ltd.) overnight at 4°C. We used goat anti-rabbit second antibody (Cell Signaling Technology Inc.) to detect protein expression and used a ChemiDoc™ MP Imaging system to visualize the target proteins (Bio-Rad Co., USA). The Image Lab software was used for analysis during the experiment. (Bio-Rad Co., USA).

### Statistical Analysis

Representation of data was expressed as the means ± standard error of mean (SEM). Meanwhile, we used SPSS software to statistically analyze the between groups made by one-way analysis of variance (ANOVA) (SPSS, USA). P values < 0.05 were identified as significant in statistics. By using GraphPad Prism 6.0, we generated all of the graphs (GraphPad, USA).

## Results

### Rhein Mitigated RSV-Caused Lung Injury and Reduced Lung Index in Mice

**Figure 2:**
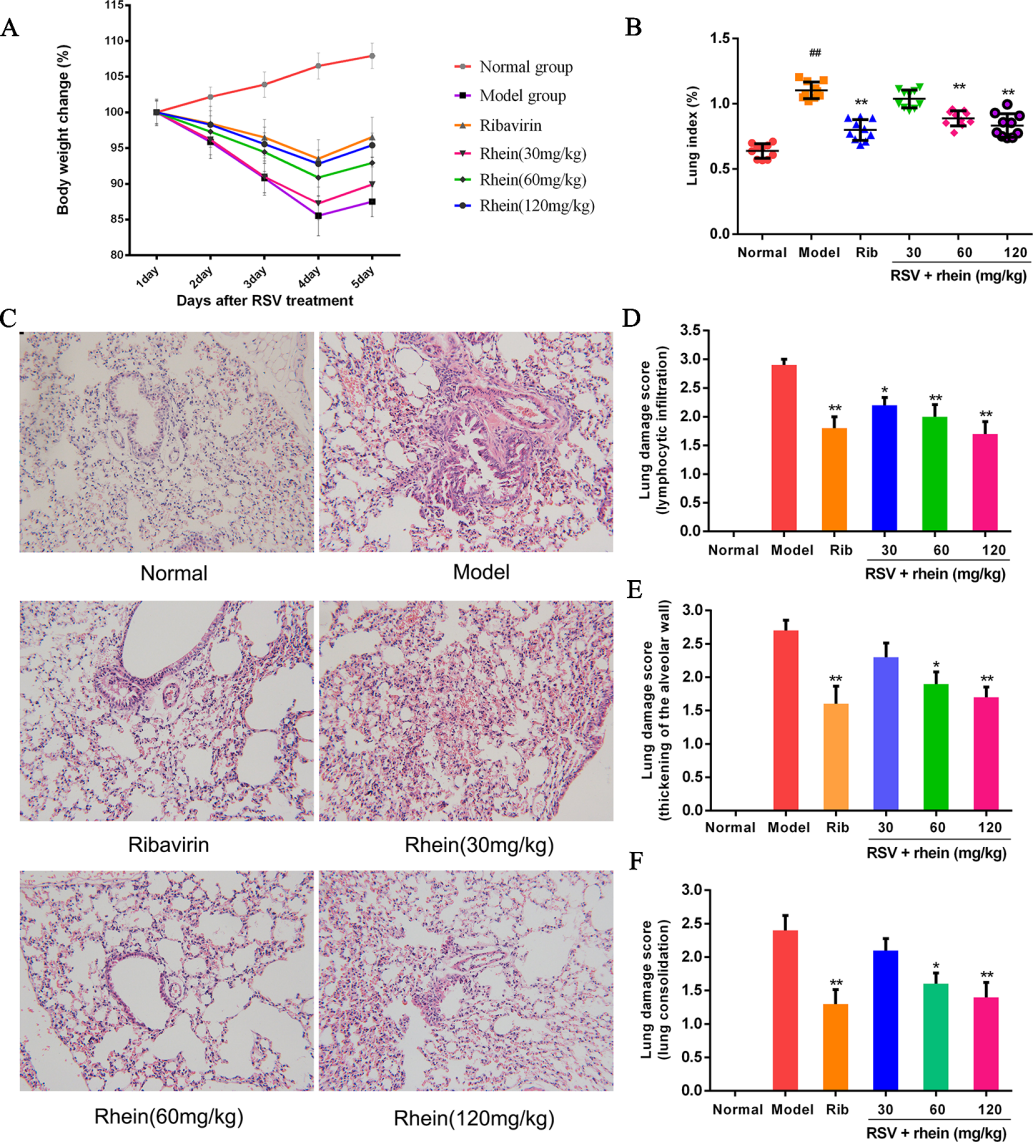
Rhein mitigated pulmonary histopathological damage and reduced lung index induced by RSV in mice. **(A)** Body weight in mice. **(B)** Lung index for RSV-caused pneumonia. **(C)** Pathological changes of lung tissue induced by RSV (200×). (1) Normal, (2) Model, (3) Ribavirin and RSV, (4) Rhein and RSV (low dose), (5) Rhein and RSV (middle dose), (6) Rhein and RSV (high dose). **(D**–**F)** Lung injury scores according to the degree of lung damage. Data were presented as the mean ± SEM, n = 10. ^##^P < 0.01 vs Normal group, *P < 0.05 and **P < 0.01 vs Model group.

The results showed that Rhein was able to alleviate RSV-induced pneumonia. Furthermore, compared with the model mice, the infected mice’s body weight was restored after the treatments (**Figure 2A**). The control group’s lung index of mice was obviously below the RSV-induced group as demonstrated in **Figure 2B**; additionally, it showed that it was successful to make the model of pneumonia caused by RSV. By using different doses of Rhein, it could dramatically reduce the lung index of mice. The therapeutic effects were evaluated by histopathology. As shown in **Figure 2C**, after severe lung inflammation and injury resulted from RSV infection, the features of infection were lymphocytic infiltration, thickening of the alveolar wall and lung consolidation. Meanwhile, the inflammatory cells infiltrated into the alveolar space and lung interstitium (Dou et al., 2013; Cai et al., 2015; Yin et al., 2016). The pathological score was assessed according to the extent of pulmonary pathological changes as **Supplementary Table 1**. As a result, after taking Rhein and ribavirin, the pathological changes of mice’s lung were relieved (**Figure 2C**), and three kinds of lung damage score were decreased (**Figures 2D**–**F**). To sum up, it turned out that Rhein was able to alleviate lung inflammation and injury effectively in RSV-induced mice.

### Rhein Reduced the IL-1β, IL-6, TNF-α, IL-18, and IL-33 Level in the Serum and Lung Tissues

**Figure 3:**
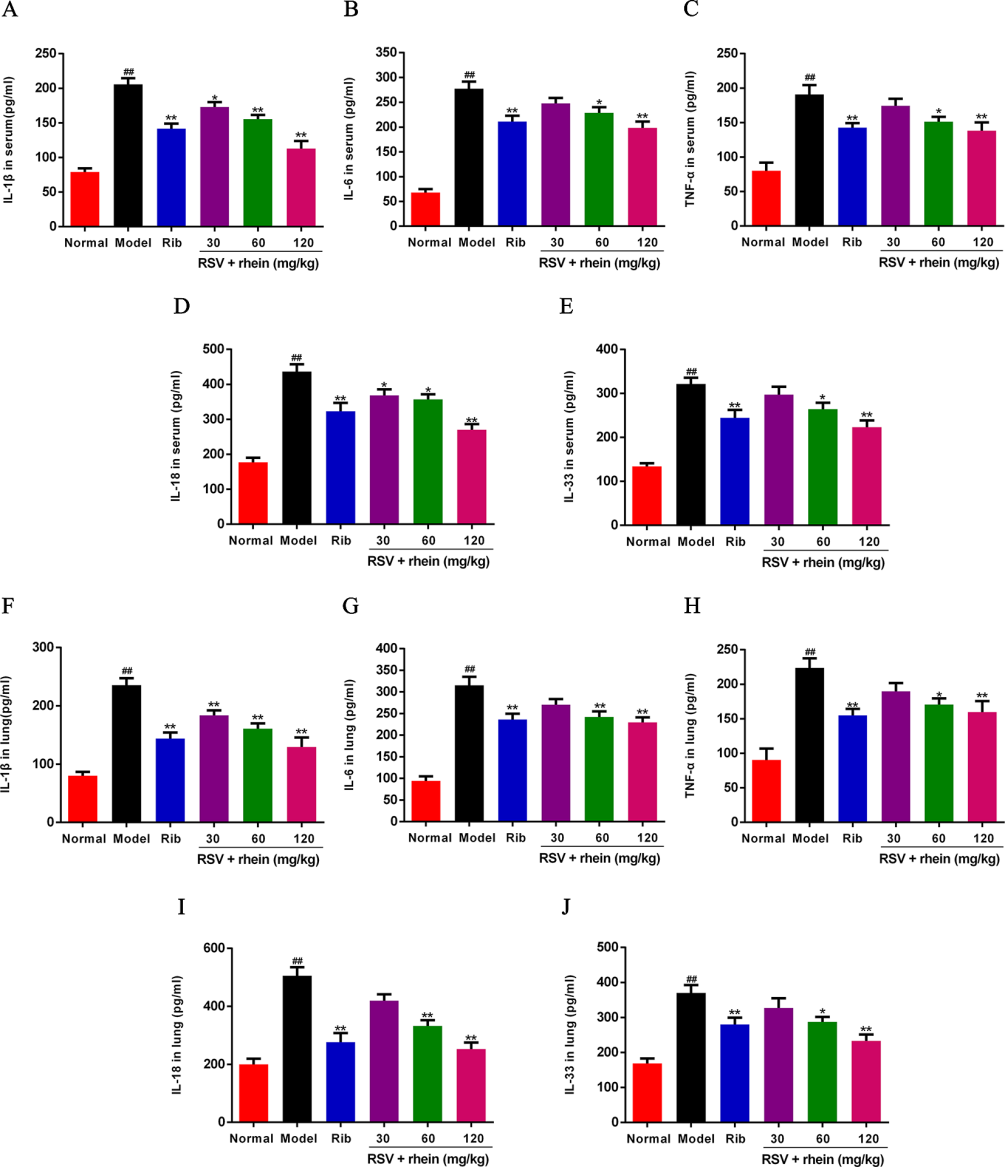
Rhein reduced the production of IL-1β, IL-6, TNF-α, IL-18, and IL-33 in serum and lung tissues of RSV-challenged mice. **(A**–**E)** Levels of IL-1β, IL-6, TNF-α, IL-33, and IL-18 in serum. **(F**–**J)** Levels of IL-1β, IL-6, TNF-α, IL-33, and IL-18 in lung tissues. Data were presented as the mean ± SEM, n = 10. ^##^P < 0.01 vs Normal group, *P < 0.05 and **P < 0.01 vs Model group.

In RSV-related damage of lung, the increase of inflammatory cytokines is one of the important pathological factors. IL-1β, IL-6, TNF- α, IL-18, and IL-33 are common inflammatory cytokines, which are involved in a series of cellular processes and eventually lead to a strong inflammatory response. In the occurrence and development of excessive inflammatory diseases induced by RSV, it is a vital function (Ma et al., 2015; Christiaansen et al., 2016; Bucher et al., 2017). Hereby, in order to confirm whether the release of inflammatory cytokines could be inhibited by Rhein, those cytokines levels in serum were detected by ELISA. As it is shown in the **Figure 3**, in the serum, the levels of TNF-α, IL-6, and IL-1β were increased effectively under the stimulus of RSV infection compared with the model group (**Figures 3A**–**C**), and Rhein was able to reduce the expressions of IL-1β, IL-6, and TNF-α effectively in a dose-dependent manner. In addition, the expressions of other IL-1 family members, in which IL-33 and IL-18 are included, presented analogous down regulation dose-dependently as well (**Figures 3D**, **E**). The changes of inflammatory cytokines in lung tissues are similar to those in serum (**Figures 3F**–**J**).

### Rhein Reduced the NLRP3 Inflammasome Activation in the Lung Tissues of RSV-Induced Mice

**Figure 4:**
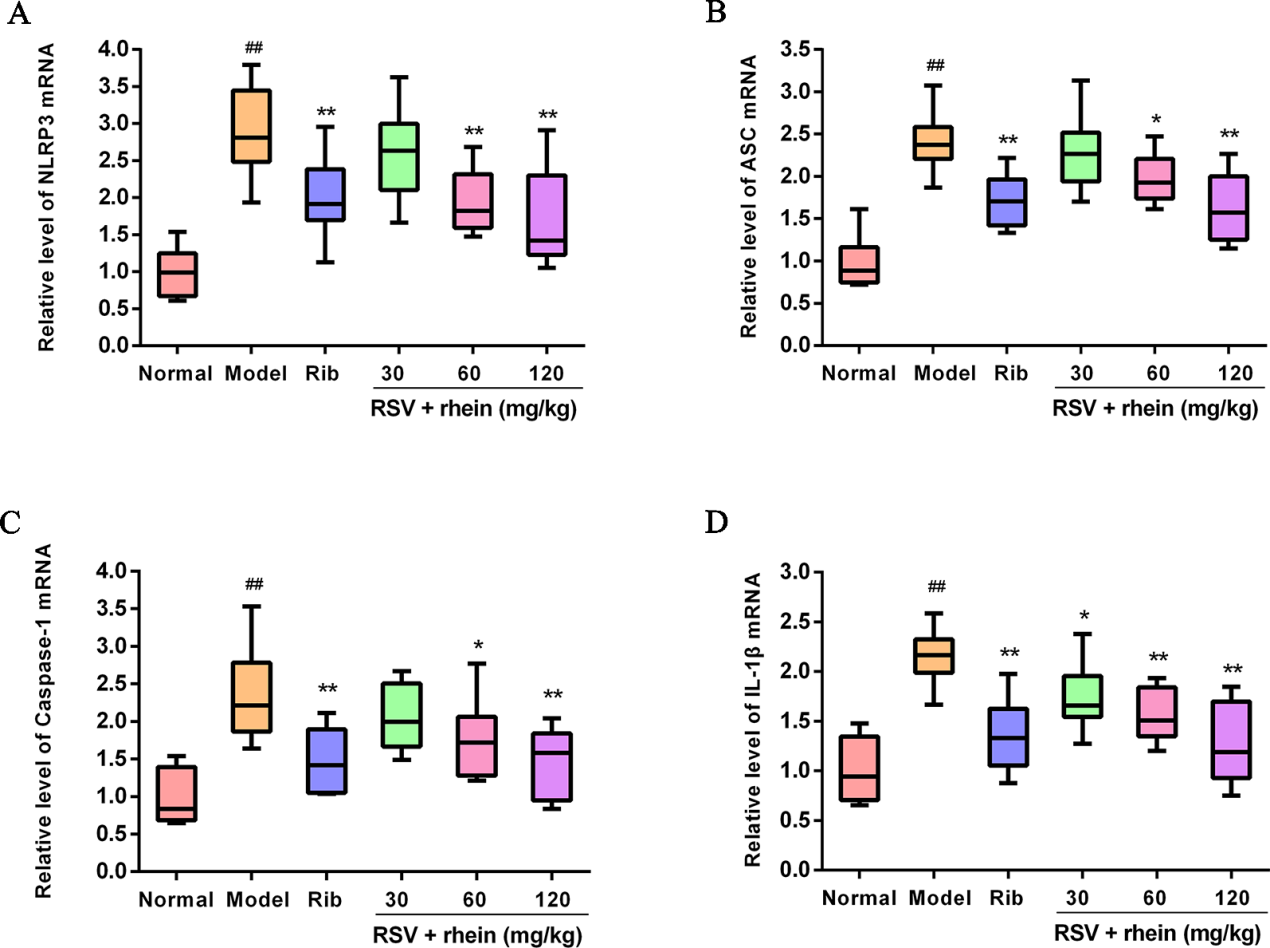
Rhein reduced the mRNA levels of NLRP3, ASC, Caspase-1, and IL-1β in the lung of RSV-challenged mice. **(A)** mRNA expression of NLRP3 was determined by Real-time PCR. **(B)** mRNA expression of ASC was determined by Real-time PCR. **(C)** mRNA expression of Caspase-1 was determined by Real-time PCR. **(D)** mRNA expression of IL-1β was determined by Real-time PCR. ^##^P < 0.01 vs Normal group, *P < 0.05 and **P < 0.01 vs Model group.

**Figure 5:**
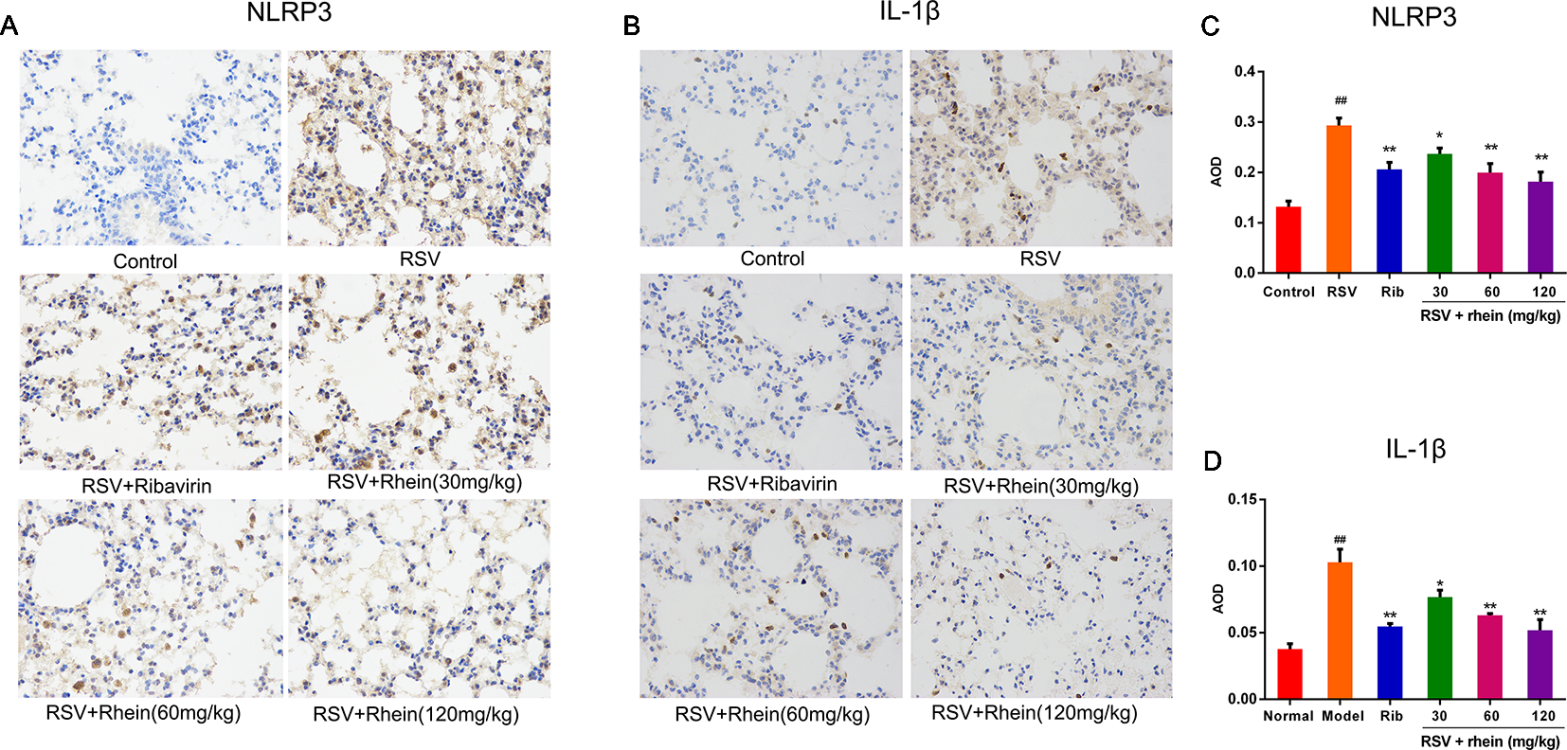
Rhein inhibited NLRP3 and IL-1β protein expression in the lung tissues of RSV-induced mice. **(A**–**B)** IHC images displaying the protein levels of NLRP3 and IL-1β in lung tissues (200×). **(C**–**D)** Protein expression of NLRP3 and IL-1β were quantified by AOD using Image-Pro Plus 6.0 software. Data were presented as the mean ± SEM, n = 10. ^##^P < 0.01 vs Normal group, *P < 0.05 and **P < 0.01 vs Model group.

**Figure 6:**
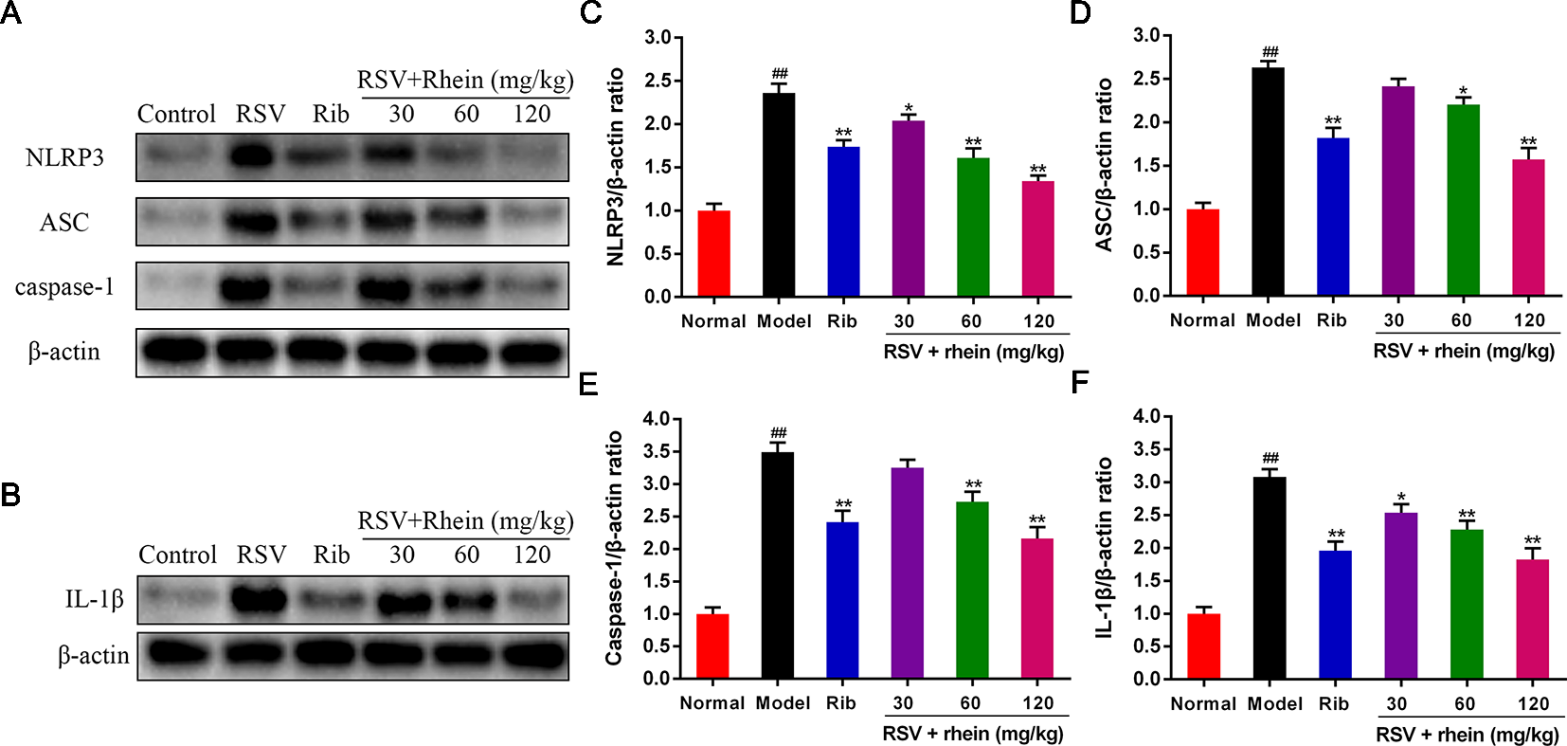
Rhein down-regulated the protein levels of NLRP3, ASC, Caspase-1, and IL-1β in the lung tissues of RSV-challenged mice. **(A)** WB determination of the protein expression of NLRP3, ASC, and Caspase-1. **(B)** WB determination of the protein expression of IL-1β. **(C**–**F)** WB quantification of NLRP3, ASC, Caspase-1, and IL-1β. Data were presented as the mean ± SEM, n = 10. ^##^P < 0.01 vs Normal group, *P < 0.05 and **P < 0.01 vs Model group.

In the process of virus infection and lung damage, the NLRP3 inflammasome pathway is very critical and is closely bound up with IL-1β, IL-33, and IL-18. It is able to convert inactive Pro-Caspase-1 into active Caspase-1 by activating NLRP3 inflammasome. Through activation by Caspases-1, Pro-IL-1β, Pro-IL-18, and Pro-IL-33 are transformed into corresponding biologically active cytokines (Triantafilou et al., 2013; Shim and Lee, 2015; Lu et al., 2017). Then, we conducted some studies to explore the effects of Rhein on these pro-inflammatory factors and whether the NLRP3 inflammasome pathway was involved in this process. Briefly, we first tested the mRNA levels of IL-1β and inflammasome-related genes NLRP3, ASC, and Caspase-1 in mice’s lung tissues using RT-PCR. In comparison with the normal control group, RSV infection (model group) was able to increase the mRNA expressions of NLRP3, IL-1β, ASC, and Caspase-1effectively as it is demonstrated in **Figure 4**. In comparison with the model group, Rhein was able to dose-dependently inhibit the mRNA transcriptions of NLRP3, IL-1β, ASC, and Caspase-1 (**Figures 4A**–**D**). Additionally, the lung tissues of mice were performed using IHC and WB assays. In the lung of RSV-challenged mice, comparison with the model group, the protein expression of NLRP3 and IL-1β in Rhein treatment groups was decreased effectively in a dose-dependent manner as it is demonstrated in **Figures 5A**–**D**. The results of WB are similar to those of IHC, as shown in **Figures 6A**–**F**. Moreover, the protein levels of ASC and caspase-1 were down-regulated to some extent as well. Collectively, our research results revealed that in mice, the excessive inflammatory response induced by RSV, which was inhibited by Rhein, may be bound up with restrain of NLRP3 inflammasome activation.

### Rhein Inhibited the NLRP3 Inflammasome Activation *via* Suppressing NF-κB Signaling in the Lung Tissues of RSV-Induced Mice

**Figure 7:**
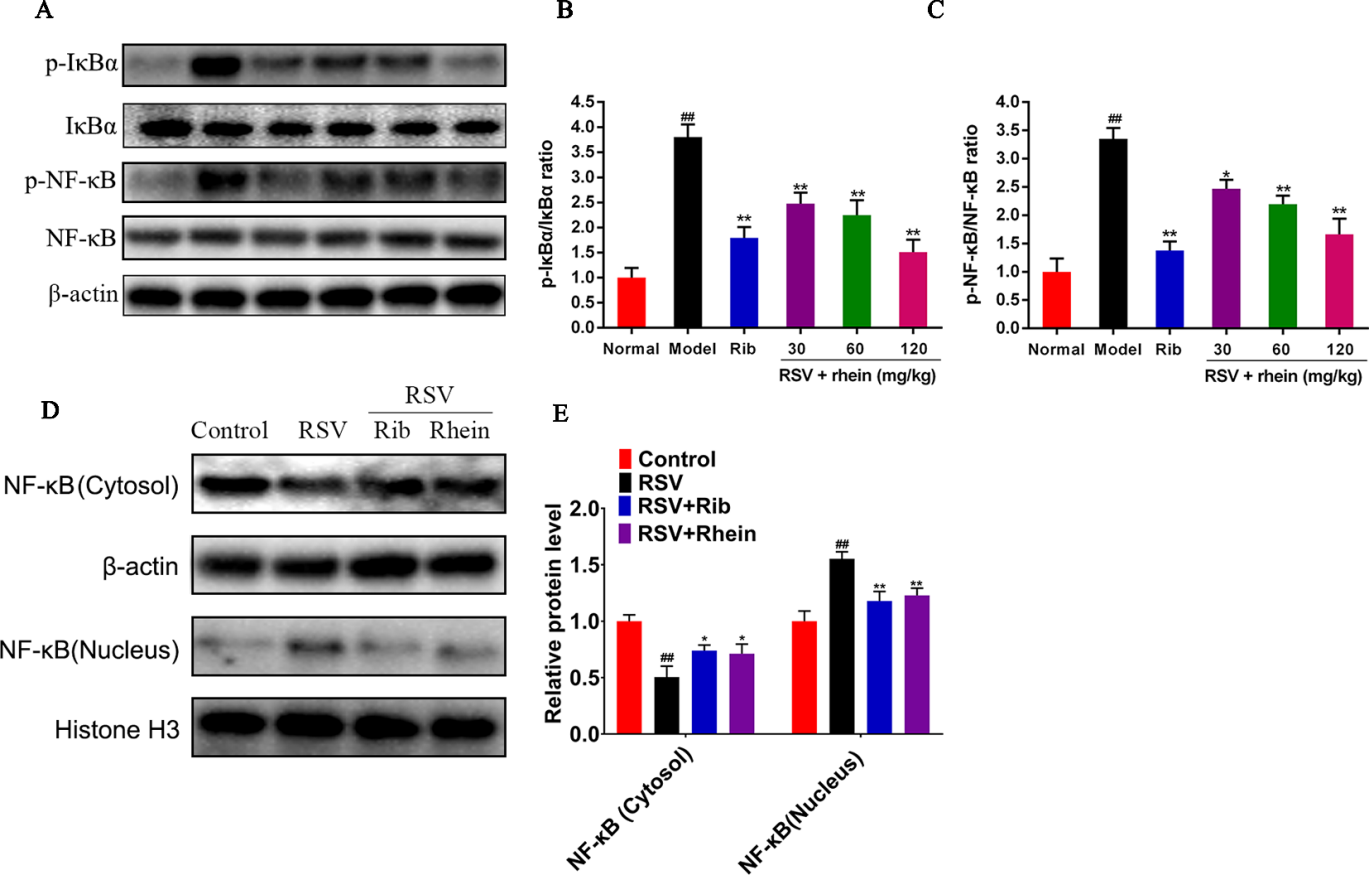
Rhein inhibited the expression of p-IκBα, p-NF-κB, and nuclear translocation of NF-κB in the lung tissues of RSV-induced mice. **(A**–**C)** WB determination of the protein expression of p-IκBα, IκBα, NF-κB, and p-NF-κB. **(D**–**E)** Protein expression of NF-κB in the Cytosol and nucleus. Data were presented as the mean ± SEM. ^##^P < 0.01 vs Normal group, *P < 0.05 and **P < 0.01 vs Model group.

Previous studies have demonstrated that NF-κB is an important factor in the activation of NLRP3 inflammasome during RSV infection and there is a positive correlation between them. Therefore, we detected the expression of NF-κB signaling pathway, and found that RSV can activate NF-κB signal, and the expression of p-IκBα, p-NF-κB is increased, while Rhein can inhibit the phosphorylation level of p-IκBα, p-NF-κB and further inhibit the entry of NF-κB into the nucleus (**Figures 7A**–**E**).

## Discussion

Nowadays, the public is paying more and more attention to traditional Chinese medicine, and the research and utilization of new drugs from traditional medicine with long-confirmed effects may be an effective treatment for a variety of diseases. RSV infection is one of the important factors of respiratory tract infection, and bronchitis and pneumonia may be resulted from it. Even though people have been struggling for years, no effective drugs to treat RSV infection were found (Jorquera and Tripp, 2017). It showed that ribavirin has anti-viral effect, and can improve pulmonary inflammation diseases resulted from RSV infection to a certain degree in prior research. Nevertheless, its adverse effects are obviously shown by prior research, too. Therefore, it is necessary to find low-toxic and effective drugs to treat excessive inflammation caused by virus.

The excessive inflammation caused by RSV infection is worthy of attention (Shi et al., 2016; Mosquera et al., 2018). There is growing evidence that certain natural products may be able to keep mice away from viral pneumonia. In previous studies, researchers pointed out that Rhein has anti-inflammatory and antiviral effects *in vivo* and *in vitro* (Chang et al., 2014; Zhou et al., 2015). Immune responses caused by RSV may lead to excessive inflammation in the airway and lung, and increase the damage of airway and lung tissue (Gu et al., 2016). To further study the underlying mechanism, the anti-inflammatory effects of Rhein in RSV- infected BALB/c mice have been investigated. The results revealed that Rhein was able to restore body weight of mice infected by RSV as well as reducing the lung index of mice and alleviating the pathological degree of pulmonary damage, including bleeding, inflammatory infiltration and thickening of alveolar wall. Those experiment results indicated that *in vivo*, Rhein was able to restrain the immune over-inflammatory responses to RSV infection.

Next, we tested the pro-inflammatory cytokines levels in lung tissues and serum of RSV-infected mice. In comparison to the RSV group, treatments with Rhein could significantly inhibit the secretion, including IL-1β and IL-6 and TNF-α, and other inflammatory cytokines IL-33 and IL-18. It is very worthwhile for us to study the potential mechanism of Rhein against RSV. In the course of virus infection, IL-1β acts as a key pro-inflammatory factor causing excessive inflammatory response, which eventually leads to tissue damage and other diseases. The NLRP3 inflammasome is one of the PRR pattern recognition receptors, and PAMP is able to activate it. NLRP3 inflammasome plays the key defense factor against invading microorganisms in the innate immune system. It is a significate molecular signal that is able to induce the release of IL-1 family members, such as IL-1β (Netea et al., 2015). The activation and subsequent processing of Caspase-1 as well as the secretion and release of cytokine IL-1 family members was regulated by NLRP3 inflammasome and the downstream associated protein ASC participating in innate immune defense (Kim et al., 2017; Poudel and Gurung, 2018). In previous researches, it was suggested that IL-1β family inflammatory elements play an important role in acute and chronic inflammation and in the meantime, NLRP3 inflammasome pathway is one of the key signaling pathways to lead to excessive inflammation (Pinkerton et al., 2017; Chen et al., 2018; Gugliandolo et al., 2018). Besides, NF-κB is a vital family of transcription factors, which is widely involved in a variety of biological processes, including inflammation. Numerous investigations have demonstrated that NF-κB is responsible for inducing NLRP3 inflammasome activation and excessive IL-1β secretion, while many types of viruses, including RSV, can activate NF-κB signal (Segovia et al., 2017; Zou et al., 2018). Our data provides compelling evidence that Rhein may ameliorate the excessive inflammation stimulated by RSV *via* interfering the NLRP3 signaling activation by NF-κB signal, effectively decrease the release of pro-inflammatory cytokines in the serum and lung tissues and the expression of p-IκB, p-NF-κB, NLRP3, ASC, Caspase-1, promote the entry of NF-κB from cytoplasm into the nucleus to stimulate the transcription of related inflammatory cytokines.

Collectively, it turns out that Rhein exerts a protective effect against the over-inflammatory response of RSV infection and reduce tissue damage *in vivo*. As to its mechanism, it is likely to reduce the release of IL-1β and its related family pro-inflammatory factors associated with the inhibition of NLRP3 inflammasome activation by NF-κB pathway. However, how to regulate the complex inflammasome pathway by Rhein need to be further investigated.

## Conclusions

To sum up, our results suggest that Rhein may be safe and effective in improving respiratory virus infection in mice. It also provides experimental basis for clinical treatment. As one of the main components extracted from *R. palmatum L*., Rhein holds the promises to become a novel potential anti-inflammatory therapeutic agent for RSV infection. Furthermore, it might be an effective way for improvement and treatment of RSV infection that inhibition of NLRP3 inflammasome may plays.

## Data Availability Statement

All datasets generated for this study are included in the article.

## Ethics Statement

Based on the National Institutes of Health Guidelines for Laboratory Animals and approved by the Animal Ethics Committee of Nanjing University of Chinese Medicine, we performed all steps of laboratory.

## Author Contributions

CS, ZZ, JS, and XZ contributed to the research idea and provided the writing and revision of the manuscript. LL, JY, and AK made contributions to the analysis of manuscripts. ZZ, TX, JJ, JX, and JY conducted the animal experiments. QD and YD contributed to the data analysis. SW and JS contributed to the constructive discussions.

## Funding

This study was financially supported by the National Natural Science Foundation of China (Grant number 81904029), (Grant number 81904254), the Natural Science Foundation of Jiangsu Province Fund (Grant number BK20151004), (Grant number BK20161573) (Grant number BK20180825). Colleges and Universities in Jiangsu Province Natural Science Research (Grant number 19KJB360010). The Project of the Priority Academic Program Development of Jiangsu Higher Education Institutions (PAPD).

## Conflict of Interest

The authors declare that the research was conducted in the absence of any commercial or financial relationships that could be construed as a potential conflict of interest.
