# Supplementary material for: Rhein Suppresses Lung Inflammatory Injury Induced by Human Respiratory Syncytial Virus Through Inhibiting NLRP3 Inflammasome Activation via NF-κB Pathway in Mice
Source: Front Pharmacol. 2020 Jan 28;10:1600. doi: 10.3389/fphar.2019.01600 (PMC6997271; doi:10.3389/fphar.2019.01600)
Supplement: Supplementary file 1 [file Table_1.docx]

**TABLE S1** Pulmonary pathology score

| [Group](file:///D:\Program%20Files\Youdao\Dict\7.5.2.0\resultui\dict\?keyword=group) | lymphocytic infiltration | thickening of the  alveolar wall | lung consolidation |
| --- | --- | --- | --- |
| Normal | 0 | 0 | 0 |
| Model | 2.9±0.32 | 2.7±0.48 | 2.4±0.69 |
| Rib | 1.8±0.63 | 1.6±0.84 | 1.3±0.67 |
| Rhein 30mg/kg | 2.2±0.42 | 2.3±0.67 | 2.2±0.63 |
| Rhein 60mg/kg | 2.0±0.67 | 1.9±0.57 | 1.6±0.52 |
| Rhein 120mg/kg | 1.7±0.67 | 1.7±0.48 | 1.4±0.69 |
